# Periodized Aerobic Training between Thresholds Improves Submaximal Cardiorespiratory Parameters in Octogenarians

**DOI:** 10.3390/sports11110219

**Published:** 2023-11-08

**Authors:** Cristian Cofre-Bolados, Félix Vidal, Héctor Gutiérrez Espinoza, Ignacio Betancourt-Peters, Pedro A. Orihuela, Mikel Izquierdo

**Affiliations:** 1 Laboratory of Sciences of Physical Activity, Sport and Health, Faculty of Medical Sciences, Universidad de Santiago de Chile, Santiago 9170022, Chile; cristian.cofre@usach.cl; 2 Navarrabiomed, Hospital Universitario de Navarra (HUN), Navarra Institute for Health Research (IdiSNA), Universidad Pública de Navarra (UPNA), 31008 Pamplona, Spain; vidal.136318@e.unavarra.es (F.V.);; 3 Escuela de Fisioterapia, Universidad de las Americas, Quito 170504, Ecuador; 4 Departamento de Ciencias Exactas, Facultad de Ingeniería, Arquitectura y Diseño, Universidad San Sebastián, Sede Tres Pascualas, Concepción 4081339, Chile; 5 Laboratorio de Inmunología de la Reproducción, Facultad de Química y Biología, Universidad de Santiago de Chile, Santiago 9160000, Chile; pedro.orihuela@usach.cl; 6 CIBER of Frailty and Healthy Aging (CIBERFES), Instituto de Salud Carlos III, 28029 Madrid, Spain

**Keywords:** older adults, octogenarians, HIIT, cardiorespiratory risk, ergometry, aerobic training

## Abstract

Background and Aims: The worldwide aging population is expanding, with more individuals living into their 80s. Physiological functions decline gradually with age, compounded by sedentary lifestyles. Incorporating physical activity into daily routine is crucial for maintaining independence. This study aimed to assess a periodized high-intensity aerobic training program (PEZO-BT) in octogenarians, focusing on submaximal ergospirometry effects. Methods: A total of 48 non-frail octogenarian subjects (12 females, 36 males) were randomized into control and intervention groups. All subjects underwent submaximal cardiopulmonary exercise testing with gas analysis at baseline, stopping after the respiratory compensation point (RCP). Our intervention group completed a 14-week PEZO-BT aerobic training program. The outcomes were oxygen consumption at first ventilatory threshold (VO_2_AT), ventilatory efficiency slope (VE/VCO_2_), oxygen uptake efficiency slope (OUES), cardiorespiratory optimal point (COP), oxygen pulse change (ΔVO_2_/HR) from anaerobic threshold (AT) to respiratory compensation point (RCP), and power output at anaerobic threshold (POAT). Results: Mixed ANOVA examined time and treatment effects. If significance emerged, post hoc t-tests were used to compare significances between groups. The homogeneity of variance was assessed using Levene’s test. Chi-square tests compared ergospirometry criteria and ventilatory performance within groups. The mean differences at post intervention were significant in VO_2_AT (*p* < 0.001), VE/VCO_2_ (*p* < 0.001), ΔVO_2_/HR (*p* < 0.05), and POAT (*p* < 0.001), while OUES and COP were not significant (*p* > 0.05). However, clinical effects were observed in the entire intervention group. Conclusions: Training improved exercise capacity and workload. Overall, this periodic aerobic and high-intensity interval training (HIIT) program yielded significant improvements in cardiorespiratory fitness (CRF) in previously untrained octogenarians with and without comorbidities. The findings suggest implications for promoting long-term healthy aging.

## 1. Introduction

Population aging is a universal process inherent to human beings. It is one of the four “mega-trends” that are shaping the current world population, along with population growth, urbanization, and international migration. Each of these mega-trends will continue to significantly impact sustainable development in the coming decades [1,2].

The world population is undergoing an epidemiological transition characterized by decreased premature mortality, increased life expectancy, and gradually declining birth rates. The octogenarian proportion has grown from 1.41% of the population in 2002 to around 2.5% a decade later [3,4]. Physiological functions decline gradually with age, and sedentary lifestyles are linked to reduced muscle function and cardiorespiratory fitness (CRF) [5,6,7], impairing the ability to perform daily activities and maintain independence [8]. However, adequate physical exercise (PE) can significantly mitigate these age-related declines in CRF [9,10]. Strategies to increase physical activity (PA) now focus on “lifestyle integration”—incorporating exercise into daily routines to make it more accessible and sustainable. The World Health Organization (WHO) recommends at least 150 min per week of moderate aerobic exercise (MAE) or 75 min of vigorous aerobic exercise (VAE) for adults over 65, plus muscle-strengthening activities at least twice weekly [11,12,13,14]. Moderate-to-vigorous aerobic exercise best improves maximal and submaximal aerobic capacity, peripheral oxygen extraction, stroke volume, and arterial stiffness [15], while high-intensity interval training (HIIT) is very effective depending on the individual’s fitness level [16,17,18,19,20,21,22,23,24,25]. These recommendations promote active, healthy lifestyles in older adults to improve quality of life and prevent chronic diseases [14]. In recent years, various training programs have emerged to be tailored to older individuals’ needs and their abilities. These programs aim to enhance overall wellbeing and functional capacity in both frail and non-frail seniors [5,9,11,13,26,27,28,29,30].

Cardiopulmonary exercise testing (CPET) assessing maximal oxygen consumption (VO_2_max) is the gold standard for measuring CRF and its clinical and sports applications [31,32,33]. Despite its potential clinical value as a cardiovascular risk factor, CRF is not routinely evaluated due to challenges, risks, costs, and selection bias [34]. Ironically, excluding higher-risk individuals masks the relationship between measured CRF and disease outcomes [35,36]. Therefore, studies have proposed submaximal CPET parameters as alternatives to maximal testing, including oxygen consumption at ventilatory threshold (VO_2_AT) [37], VE/VECO_2_ slope (ventilatory class) [35], oxygen uptake efficiency slope (OUES) [38], cardiorespiratory optimal point (COP) [39,40] or minute ventilation equivalents [41,42], and oxygen pulse (VO_2_/HR) at anaerobic threshold (AT) and respiratory compensation point (RCP) [7,27,43]. Defined cutoffs distinguish normal from abnormal CPET results [7,44]. Additionally, submaximal continuous exercise (SCE) improves respiratory strength, lung function, exercise tolerance, fatigue, and quality of life [45,46,47].

This study will assess a periodized high-intensity aerobic training program (PEZO-BT) in octogenarians, focusing on submaximal ergospirometry effects.

## 2. Materials and Methods

### 2.1. Subjects

A total of 48 octogenarian adults, including both men and women, were included in the study through a convenience sampling method (Table 1). Participants were individuals who successfully completed the intervention, demonstrating over 80% attendance across all scheduled sessions (14 weeks of training, 3 sessions per week). This cohort was selected from an initial pool of 54 older adults. The inclusion criteria required participants to be over 80 years old at the outset of the study, have stable cardiometabolic pathologies as determined by medical assessment, and pass the initial medical examination, which included a resting ECG, anamnesis, and the same submaximal ergometry.

They were selected for their participation in PA programs specifically tailored for older adults. These programs were conducted by the Young Men’s Christian Association (YMCA) in Santiago, Chile, with a focus on their enrollment in the Center for Adapted Exercise (CAE) program. This program was designed to assist older adults in resuming exercise after an 18-month period of inactivity resulting from the SARS-CoV-2 pandemic. All participants were assessed as functionally independent and devoid of frailty, having previously engaged in physical exercise programs at the YMCA in Santiago before the pandemic.

Throughout the confinement period, which was characterized by limited physical activity opportunities, none of the participants engaged in structured or supervised exercise sessions. Initial activity, prior to resuming their exercise program, involved a submaximal CPET evaluation to guide their subsequent training regimen based on the assessment outcomes. The subjects were randomly assigned into control (23) and intervention (25) groups. The control group performed concurrent training sessions starting and finishing with 10 min of continuous aerobic training ranging from 50 to 60% of their heart rate reserve (HRR). Strength training was arranged in circuits of 5 exercises (leg press, leg extension, chess press, lat row, and femoral curl) performed on the same day, completing 3 sets of 10 repetitions at 50 to 60% of a repetition maximum (RM) for each exercise, and resting 2 min between sets and 3 to 5 min after completing one lap of the circuit. The intervention group performed a PEZO-BT program. The control and intervention groups trained 3 times per week for a 14-week period. In addition, aerobic training was monitored with a heart rate monitor (Polar M430). The subjects were supervised by a qualified sports scientist coach at CAE facilities.

Informed consent was obtained from all participants, and the study protocol was approved by the Ethics Committee of the University of Santiago. This research adhered to the principles outlined in the Declaration of Helsinki.

### 2.2. Submaximal CPET Test

**Figure 1:**
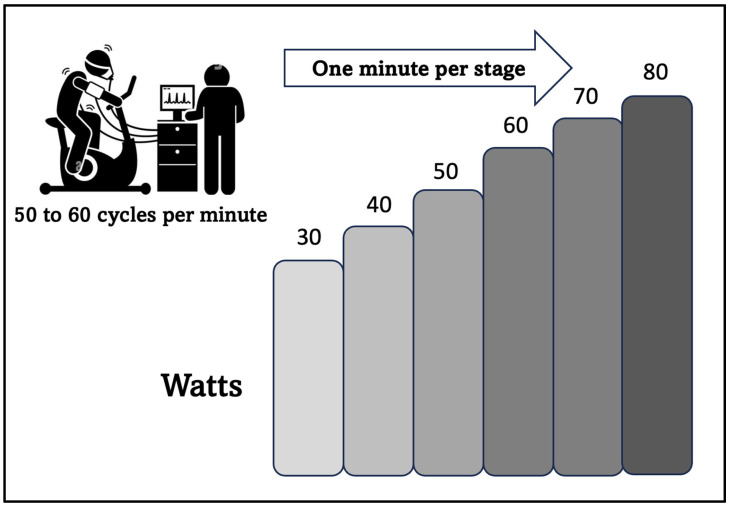
Ramp protocol with a 10-watt load increase per minute, starting at 30 watts (aimed at elderly population with low aerobic power).

The participants underwent a preventive and pre-participation medical evaluation conducted by specialist physicians in Sports Medicine and Physical Activity. This evaluation included the measurement of health parameters such as resting heart rate, blood pressure, and overall musculoskeletal status for the test. Subsequently, they underwent a submaximal CPET using a Cortex Metamax 3B gas analyzer, followed by an adapted incremental protocol performed on a Technogym Excite _(MR)_ cycle ergometer. The test started with an initial load of 30 watts, with 10-watt increments every minute, maintaining a cadence between 50 and 60 cycles per minute (Figure 1), and concluded upon reaching the RCP, determined through graphs 6 and 9, according to the nine-panel proposal by Wasserman (ventilatory equivalents and end-tidal pressures of oxygen and carbon dioxide, respectively). These results allowed us to determine the AT and RCP, specifically using graphs 6 and 9 by Wasserman, as recommended by the equipment manufacturer, along with confirmation by a second expert. Additionally, reference submaximal parameters associated with diagnosis, prognosis, and life expectancy were recorded, including VO_2_AT, OUES, VE/VCO_2_, COP, and ∆VO_2_/HR (RCP vs. AT).

### 2.3. Training Zones

**Figure 2:**
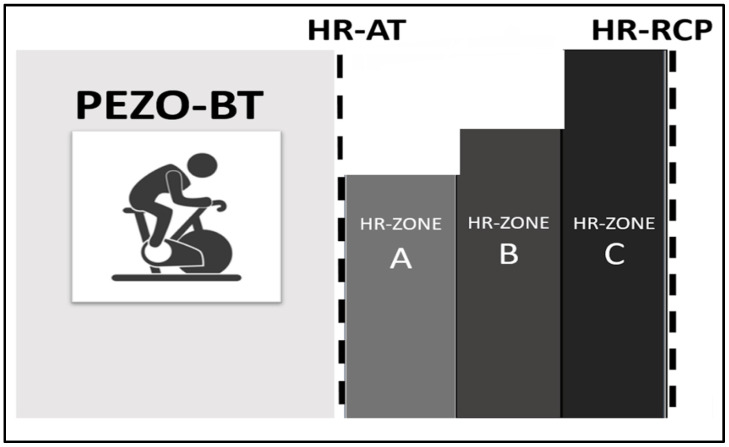
Training zones between thresholds. The aerobic training PEZO-BT used the individuals’ HR established for ZONE A, B, and C.

The determined thresholds were used to define PEZO-BT for each participant to implement a progressive and individualized training program. This program encompasses the continuous aerobic (CA) method, the stepped or incremental aerobic (IA) method, and the HIIT method, divided into three phases based on the subdivision of three zones between the thresholds: Zones A, B, and C [16,17,20,48,49,50]. Each third is stated by heart rate to control the intensity of every period (Figure 2).

### 2.4. Training

During the first period (mesocycle of adaptation), a CA training was conducted for 6 weeks. This involved cycle ergometer sessions at Zone A intensity, with a gradual progression of duration: 10 to 15 min in the first 2 weeks, 15 to 25 min in the following 2 weeks, and finally 25 to 30 min in the last 2 weeks. These sessions were performed 3 times per week.

In the second period (mesocycle of development), lasting 4 weeks, the IA method was implemented. Different intensity zones were alternated during 20 and 25 min of cycle ergometer sessions. For example, during the first 2 weeks, there were intervals of 5 min at Zone A intensity, followed by 5 min at Zone B intensity, and then again at Zone A and B. In the next 2 weeks, the intervals were 5 min at Zone A, 5 min at Zone B, 5 min at Zone C, and again at Zone A. These sessions were performed 3 times per week.

**Figure 3:**
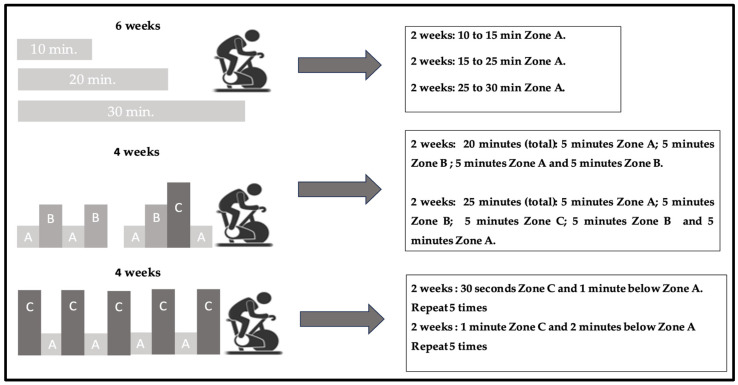
Overview of the training program of Periodization Zone between Thresholds (PEZO-BT).

The third period (mesocycle of shock) lasted 4 weeks and involved the HIIT method. The sessions were performed at Zone C intensity and recovery at Zone A intensity. During the first 2 weeks, there were intervals of 30 s at Zone C, followed by 1 min below Zone A, repeated 5 times. In the next 2 weeks, the intervals were 1 min at Zone C, followed by 2 min below Zone A, also repeated 5 times. These sessions were conducted 2 times per week, with a minimum rest period of 72 h (Figure 3).

### 2.5. Statistical Analysis

Statistical analyses were performed using R (4.1.0), to test the hypotheses of interest. A mixed ANOVA analysis was considered, with time as the within-subject factor, considering 2 levels (2 measurements, pre and post), and treatment (group type) as the between-subject factor, which also had 2 levels (control group and experimental group). It was examined whether the interaction effects, and then the main effects, were significant according to the ANOVA analysis. The main effects of both the treatment and time allowed us to assess if there was an influence of these factors on the dependent variables, while the interaction effect was associated with whether the influence of one factor depended on the level of the other. The size of the effect (SE) associated with each effect was obtained through the results of eta squared. Values between 0.01 and 0.059 are considered small effect, values between 0.06 and 0.0139 are considered medium effect, and values over 0.14 are considered large effect.

If significant effects were found, post hoc tests of means (t-tests) were subsequently conducted, comparing the levels of their respective factors, and exploring the differences. Bonferroni correction was applied for significance, with a significance level set at 5%. Prior to analysis, the homogeneity of variances across groups was checked using Levene’s test separately for the pre and post time points. Additionally, a chi-square test, considering Yates’ continuity correction, was performed to compare ergospirometry criteria and ventilatory performance within the control and intervention groups based on contingency tables.

## 3. Results

All subjects completed the 14-week intervention, and all subjects of the control and intervention groups were evaluated. The results are presented in box plots to display the minimum, first quartile, median, third quartile, and maximum values of the submaximal variables of the subjects. Also, a contingency table was used to investigate the presence of an association or independence between variables by comparing the observed frequencies with the expected frequencies (Table 2). This analysis facilitated the classification of subjects as either normal or altered, along with the assignment of their corresponding performance scores.

### 3.1. Ergospirometry Submaximal Variables

**Figure 4:**
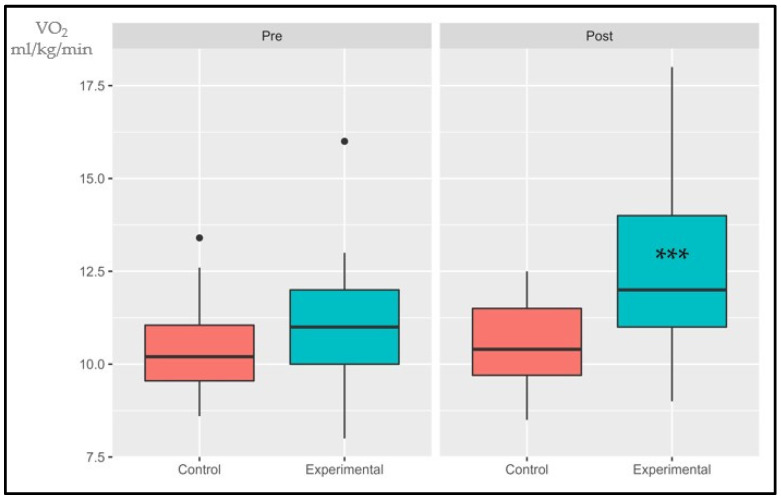
Absolute changes in VO_2_ at AT after 14 weeks of intervention; ● represents outlier data. Values are expressed in mL/kg/min. *** *p* < 0.001.

**Figure 5:**
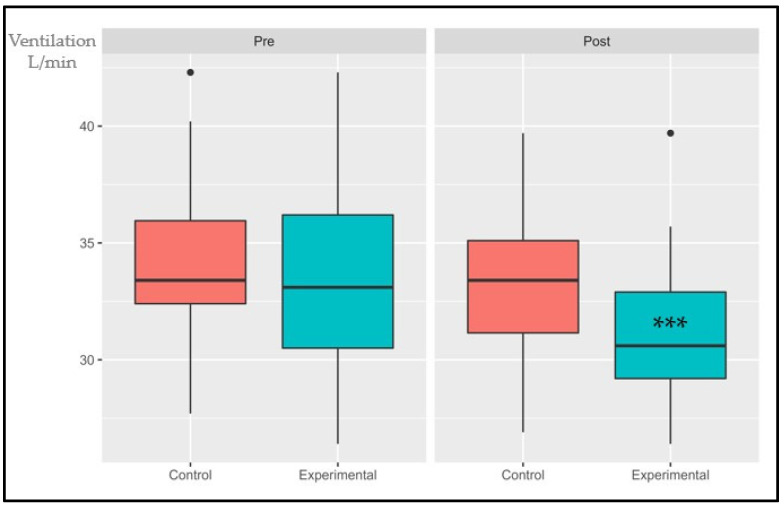
Absolute changes in ventilatory class after 14 weeks of intervention; ● represents outlier data. Values are expressed in L/min. *** *p* < 0.001.

**Figure 6:**
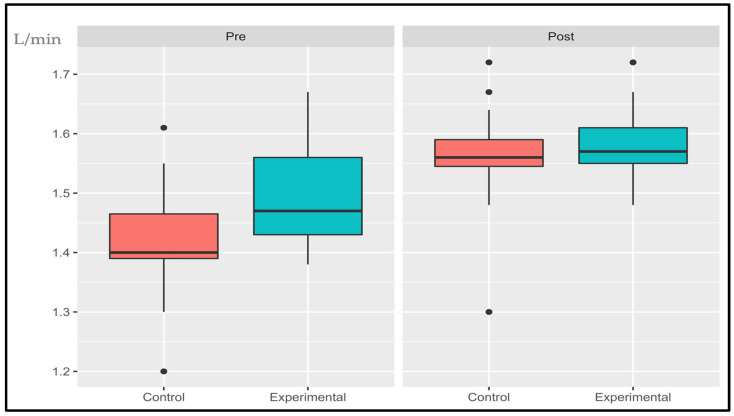
Absolute changes in OUES after 14 weeks of intervention; ● represents outlier data. Values are expressed in L/min.

**Figure 7:**
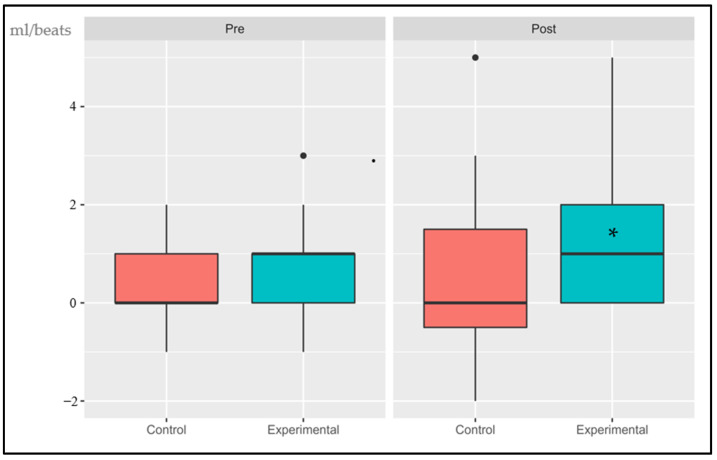
Absolute changes in ∆VO_2_/HR (RCP vs. AT) after 14 weeks of intervention; ● represents outlier data. Values are expressed as the difference in mL-beats of VO_2_/HR at RCP and AT. * *p* < 0.05.

**Figure 8:**
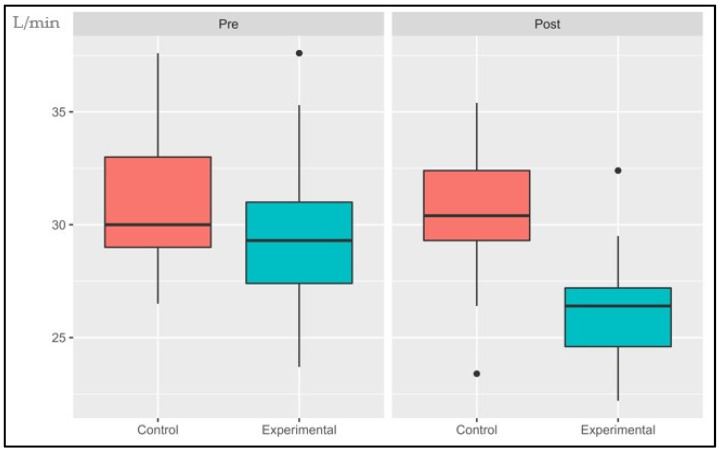
Absolute changes in COP after 14 weeks of intervention; ● represents outlier data. Values are expressed in L/min.

**Figure 9:**
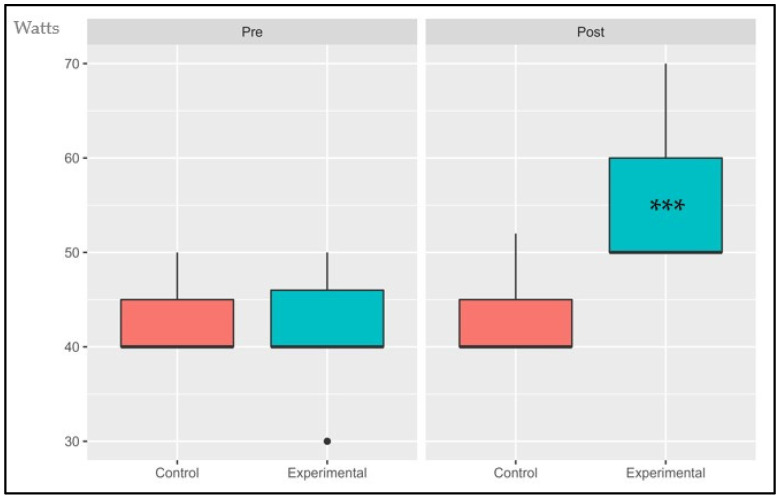
Absolute changes in power output after 14 weeks of intervention; ● represents outlier data. Values are expressed in Watts. *** *p* < 0.001.

The following data are presented in box plots (Figure 4, Figure 5, Figure 6, Figure 7, Figure 8 and Figure 9), where the interested variables were analyzed. The control and experimental groups were compared in the pre and post period. 

#### 3.1.1. VO_2_AT

The interaction effect, primary effects of treatment, and time were significant: (*p* < 0.001, SE = 0.04) and (*p* = 0.006, SE = 0.145); (*p* < 0.001, SE = 0.055), respectively. There were no statistical differences in the pre period (*p* = 0.155) while the post period showed significant differences (*p* < 0.001). The control group had an average increase of 1.67% while the experimental group had an increase of 14.4%.

#### 3.1.2. Ventilatory Class (VE/VCO_2_)

The interaction effect and primary effects of the treatment were not significant: (*p* > 0.05, SE = 0.007) and (*p* > 0.05, SE = 0.05), respectively. Time showed significant changes (*p* < 0.001, SE = 0.056). There were no statistical differences in the pre period (*p* = 0.0572) while the post period show significant differences (*p* < 0.001). The control group had an average decrease of 2.95% while the experimental group had a decrease of 6.39%.

#### 3.1.3. Oxygen Uptake Efficiency Slope (OUES)

The interaction effect and primary effects were not significant: (*p* > 0.05, SE = 0.054) and (*p* > 0.05, SE = 0.102), respectively. However, time showed significant changes (*p* < 0.001, SE = 0.361). There were no statistical differences in the pre or post period: (*p* = 0.789) and (*p* = 0.636), respectively. The control group had an average increase of 11.15% while the experimental group had an increase of 5.48%.

#### 3.1.4. Delta Oxygen Pulse (∆VO_2_/HR RCP vs. AT)

The interaction effect, the primary effects, and time were not significant: (*p* > 0.05, SE = 0.013), (*p* > 0.05, SE = 0.102) and (*p* < 0.001, SE = 0.037), respectively. There were no statistical differences in the pre period (*p* = 0.1) while the post period showed significant changes (*p* < 0.05). The control group had an average increase of 1.93% while the experimental group has an increase of 9.85%.

#### 3.1.5. Cardiopulmonary Optimal Point (COP)

The interaction effect was significant (*p* < 0.05, SE = 0.083). The primary effect was not significant (*p* > 0.05, SE = 0.218), while time showed significant changes (*p* < 0.001, SE = 0126). There were no statistical differences in the pre or post period: (*p* = 0.622) and (*p* = 0.347), respectively. The control group had an average decrease of 1.08% while the experimental group had a decrease of 12.15%.

#### 3.1.6. Power Output at AT (POAT)

The interaction effect, the primary effects, and time showed significant changes: (*p* < 0.001, SE = 0.260), (*p* = 0.006, SE = 0.181) and (*p* < 0.001, SE = 0.260), respectively. There were no statistical differences in the pre period (*p* = 1.4) while the post period presented statistical differences (*p* < 0.001). The control group had an average decrease of 0.62% while the experimental group has an increase of 31.6%.

### 3.2. Contingence Table

Comparison of ergospirometry criteria and ventilatory performance within the control and intervention groups.

## 4. Discussion

No previous study has examined the impact of a periodized aerobic endurance training approach within the PEZO-BT zone on CRF, determined by analyzing submaximal parameters during CPET, stopped at the RCP in elderly individuals of both sexes with comorbidities. Here, we show that 14 weeks of CA, combining IA with integrated HIIT, yielded positive changes in CRF. This was evidenced by enhancements in VO_2_AT, VE/VCO_2_, OUES, COP, ∆VO_2_/HR RCP vs. AT, and POAT, providing a potential strategy to counteract established age-related physiological declines in untrained octogenarians.

CRF can be assessed using submaximal parameters with comparable predictive strength for morbidity and mortality as VO_2_max [7,40,47,49,51,52,53,54]. Previous studies [55] reported that short-term, low-volume aerobic training with HIIT (2–8 weeks) improved peakVO_2_ (~4–13%) in sedentary and recreationally active healthy adults, while a 4-week HIIT program increased peak VO_2_ (9.6%) and anaerobic threshold (13%) in healthy older adults. Similarly, in our study, VO_2_AT increased by 14%. Values below 11 mL/kg/min are considered high-risk for surgical mortality [37], suggesting potential broader implications for healthy aging, as improved CRF is associated with lower all-cause mortality and cardiovascular events. Although not all subjects in the intervention group showed significant changes, there were notable improvements in their performance scores. The percentage of individuals with no limitation increased from 8% to 60%, those with moderate limitation decreased from 64% to 36%, and those with severe limitation decreased from 28% to 4% (*p* < 0.05). In contrast, the control group exhibited no change in individuals with no limitation (0% both pre and post assessment), an increase from 47.8% to 69.5% in those with moderate limitation, and a decrease from 52.2% to 30.4% in individuals with severe limitation (*p* < 0.05).

The VE/VCO_2_ slope reflects exercise tolerance and predicts mortality in heart failure patients [56]. Regular endurance training can improve ventilatory efficiency during exercise, potentially by reducing peripheral chemoreceptor sensitivity and lowering the VE/VCO_2_ slope [57]. Slope values exceeding 34 are associated with an increased risk of mortality [58]. In our study, the VE/VCO_2_ slope significantly decreased from 34.4 to 33.25, falling below the slope threshold of 34. Initially, nine subjects had an abnormal VE/VCO_2_ slope, but after the training period, only three retained this abnormality. Recently, the VE/VCO_2_ slope has been proposed as an alternative assessment for research involving individuals with health- or age-related limitations [59].

A meta-analysis in heart failure patients with preserved ejection fraction found that HIIT did not result in improvements in VE/VCO_2_ slopes [60], which contrasts with our findings. It is important to note that exercise duration varied across studies, which could potentially influence outcomes [61,62]. In our experimental group, the percentage of individuals with an abnormal slope decreased from 36% to 12%. In a 12-week study on HIIT that examined improvements in mitochondrial bioenergetics among heart failure patients, pronounced increases in OUES were observed after the intervention. This variable represents the relationship between ventilation per minute and oxygen uptake during incremental exercise, encompassing both respiratory function and musculoskeletal performance within a single index. In contrast, the control group received only medical counseling without exercise [63]. However, it is important to note that our study did not find a significant difference in OUES changes post training between the experimental and control groups. OUES represents total body efficiency providing a comprehensive view of physiological efficiency, not limited to peripheral efficiency alone. In contrast to the VE/VCO_2_ ratio, which primarily considers pulmonary conditions, this distinction highlights the differences between these two variables [7].

The oxygen pulse serves as a metabolic surrogate for stroke volume, which typically increases and then stabilizes during exercise. Premature flattening of the VO_2_/HR curve and a decrease in the oxygen pulse in later stages of exercise indicate inadequate cardiac performance relative to increased effort demands [64]. In a study of U.S. veterans, changes in the oxygen pulse occurred concurrently with alterations in peripheral endothelial function and smooth muscle activity following an aerobic training program [65]. These findings align with our study, where the intervention group improved the oxygen pulse delta between CRP and AT by almost 10% (9.85%), while the control group improved by only 1.85%. The development of myocardial ischemia during exercise may result in a reduction in stroke volume, leading to premature flattening of the O_2_ pulse curve. A descending O_2_ curve has been suggested to indicate the presence of exercise-induced myocardial ischemia, assessed through myocardial perfusion imaging [66], thus the association between myocardial fibrosis and an anomaly in the O_2_ pulse has also been demonstrated [67]. Given the potential presence of many of the pathological conditions studied and their association with older populations, particularly octogenarians, the delta in VO_2_/HR between RCP and AT has been utilized. The absence of a difference in favor of the RCP value or even a drop in the oxygen pulse at RCP vs. AT is considered abnormal [68].

COP represents the integration of the circulatory and pulmonary systems [69]. A study involving 3331 individuals defined COP categories with cutoffs: <22 L/min, 22–30 L/min, and >30 L/min. COP > 30 L/min strongly and independently predicted all-cause mortality, either alone or in conjunction with lower VO_2_max [39]. Following the intervention, our experimental group had fewer subjects with COP alterations (reducing from 11 to 4), whereas the control group experienced an increase (from 11 to 13) in individuals with COP > 30 L/min. An earlier study [40] delineated that a high COP, either independently or in conjunction with VO_2_ max, could serve as a robust predictor for all-cause mortality in community-residing adults, whether apparently healthy or afflicted with chronic conditions. COP, a submaximal prognostic index, offers a convenient adjunct to CPET assessment, particularly valuable for individuals unable or unwilling to attain maximal exertion, as observed in this investigation. Consequently, the COP index within the intervention group exhibited a substantial reduction from an initial 44% impairment (11 subjects) to a mere 4% (1 subject) post-intervention.

Furthermore, we have integrated POAT as an indicator of mechanical efficiency, concomitant with enhancements in the submaximal parameters. A meta-analysis [70] ascertained that variables such as VE/VCO_2_ slope, OUES, maximal oxygen pulse, and respiratory exchange ratio (RER) responded comparably to both MAE and HIIT. These findings align with a systematic review encompassing patients with coronary disease, both with and without reduced ejection fraction [71].

As far as we know, no prior study has examined the effects of an octogenarian training program on these six selected submaximal parameters. We report that 14 weeks of training had a positive influence on submaximal parameters, likely through cardiorespiratory and mitochondrial bioenergetic adaptations explained by the Wasserman model [33]. Additionally, our findings suggest specific or integrated systemic adaptations of the respiratory, cardiovascular, and muscular systems, as represented in the theoretical model adaptation we propose. This adaptation aims to elucidate the interdependence of muscle tissue, the heart, the lungs, and their respective physiological systems, with each of the selected parameters.

Our findings provide a potential strategy to counteract the physiological age-related declines established in octogenarians with illnesses, aiming to restore parameters within normal ranges and consequently promote efficient individual and integrated functioning of the respiratory, cardiovascular, and muscular systems, as postulated by the model. This would constitute a theoretical contribution from our study.

A specific program of continuous aerobic training in the VO_2_AT zone [51] and HIIT [72] in the CRP zone has been shown to increase submaximal parameters, offering numerous benefits for cardiorespiratory fitness and overall health. The combination of both methods and their physiological adaptations enhances the overall effectiveness of the training program. Our study reveals that the training program increases VO_2_AT and improves other submaximal parameters, along with exercise workload capacity.

Previous studies have demonstrated that short-term, 2- to 8-week HIIT programs effectively increased peak VO_2_ (by approximately 4–13%) in sedentary and recreationally active adults [55,73,74]. These findings hold significant implications for healthy aging, as improved cardiorespiratory fitness is associated with reduced cardiovascular and all-cause mortality, along with enhancements in cardiorespiratory components [75,76].

However, there are limitations to this study. Firstly, the sample size was relatively small. Future research should consider larger sample sizes, as a larger sample may provide a more accurate representation of the differences between the control and experimental groups, especially for variables that did not change significantly during the 14-week intervention. Secondly, we did not control for the nutritional and dietary status of the participants, which could potentially influence the impact of training. Factors such as digestive pathologies, calorie intake, protein percentage, and hydration status should be considered in future investigations.

## 5. Conclusions

This study examined the effects of a 14-week periodized aerobic training program that incorporated HIIT on octogenarians of both sexes with comorbidities. The training had a positive impact on submaximal cardiopulmonary exercise test parameters, including oxygen consumption at the ventilatory threshold, slope of ventilatory efficiency, slope of oxygen absorption efficiency, cardiorespiratory sweet spot, oxygen pulse change from anaerobic threshold to respiratory compensation point, and output power at anaerobic threshold. These improvements likely resulted from cardiorespiratory and mitochondrial adaptations, providing a strategy to counteract age-related physical declines. The training increased exercise capacity and workload. Overall, this periodic aerobic program and HIIT produced significant improvements in cardiovascular fitness in previously untrained octogenarians with and without comorbidities. Our findings suggest implications for promoting long-term healthy aging.

## 6. Practical Applications

This study was not conducted at our laboratory facilities; instead, it was performed and controlled at an exercise center dedicated to elderly individuals (CAE), where the subjects trained daily. This demonstrates the real-world applicability of our PEZO-BT program. We utilized ergospirometry to determine the ventilatory thresholds, but it is worth noting that low-cost equipment and alternative methods, such as lactate curve analysis or the talk test, are currently available for threshold determination.

## Figures and Tables

**Table 1:** Characteristics (*n* = 48) of the octogenarian sample.

Variable	Group	
	Control (8M; 15F)	Intervention (4M; 21F)
Age (y), mean ± SD	83.4 ± 3.5	83.3 ± 3.4
Weight (kg), mean ± SD	64.9 ± 11.1	66.4 ± 13.7
Height (m), mean ± SD	1.58 ± 7.1	1.58 ± 7.4
BMI (kg/m^2^), mean ± SD	25.9 ± 7	26.4 ± 4.7
Cardiometabolic disease, number (%)	16 (68)	18 (72)
Cardiometabolic drugs, number (%)	14 (61)	16 (64)

Values are presented as mean and standard deviation. Percentages of both groups that present with controlled, non-complex cardiometabolic diseases, such as arterial hypertension, diabetes mellitus, insulin resistance, dyslipidemia, or fatty liver, are included, in addition to the percentage of subjects in the sample who use cardiometabolic drugs. (Data extracted from the medical file of each participant).

**Table 2:** The table shows the clinical differences between the two groups, classifying the variables as either normal or altered.

		Group Differences							
Variable	Category	Control		χ^2^	*p*	Intervention		χ^2^	*p*
		Pre Mean (SD)	Post Mean (SD)			Pre Mean (SD)	Post Mean (SD)		
**VO_2_AT (mL/kg/min)**	<11 altered	17 (73.9)	16 (69.5)	0	0.99	10 (40)	4 (16)	4.477	0.034
	≥11 normal	6 (26.1)	7 (30.5)			15 (60)	21 (84)		
**VE/VCO_2_ (L/min)**	>34 altered	9 (39)	8 (34.7)	0	0.99	9 (36)	3 (12)	3.315	0.069
	≤34 normal	14 (61)	15 (65.3)			16 (64)	22 (88)		
**OUES (mL/min)**	<1550 altered	21 (91.3)	6 (26)	0.01	0.971	15 (60)	6 (24)	3.299	0.069
	≥1550 normal	2 (8.7)	17 (74)			10 (40)	19 (76)		
**COP (L/min)**	>30 altered	11 (47.8)	13 (56.5)	0	0.99	11 (44)	1 (4)	0.015	0.902
	≤30 normal	12 (52.2)	10 (43.5)			14 (56)	24 (96)		
**∆VO_2_/HR RCP vs. AT**	<0 altered	14 (61)	13 (57)	0.256	0.613	9 (36)	7 (28)	0	0.99
	≥0 normal	9 (39)	10 (43)			16 (64)	18 (72)		

VO_2_AT (mL/kg/min), VO_2_ at AT; VE/VCO_2_ (L/min), ventilatory class (L/min); OUES, oxygen uptake efficiency slope (mL/min); COP, cardiorespiratory optimal point(L/min); ∆VO_2_/HR RCP vs. AT, difference between oxygen pulse at RCP and AT. The submaximal variables are categorized as altered or normal according with their cutoff points. The ventilatory performance score was derived from the sum of abnormal criteria of each submaximal variable.

## Data Availability

The datasets generated and/or analyzed during the current study are not publicly available due the terms of consent/assent to which the participants agreed but are available from the corresponding author on reasonable request. Please contact the corresponding author to discuss availability of data and materials.
